# Collaborative Intelligence to catalyze the digital transformation of healthcare

**DOI:** 10.1038/s41746-023-00920-w

**Published:** 2023-09-25

**Authors:** Ami B. Bhatt, Jennifer Bae

**Affiliations:** 1 Chief Innovation Officer, American College of Cardiology, Associate Professor, Harvard Medical School, Washington, USA; 2 Executive Director of Global Innovation, American College of Cardiology, Washington, USA

**Keywords:** Health care, Health occupations

## Abstract

Collaborative intelligence reflects the promise and limits of leveraging artificial intelligence (AI) technologies in clinical care. It involves the use of advanced analytics and computing power with an understanding that humans bear responsibility for the accuracy, completeness and any inherent bias found in the training data. Clinicians benefit from using this technology to address increased complexity and information overload, support continuous care and optimized resource allocation, and to enact efforts to eradicate disparities in health care access and quality. This requires active clinician engagement with the technology, a general understanding of how the machine produced its insight, the limitations of the algorithms, and the need to screen datasets for bias. Importantly, by interacting, the clinician and the analytics will create trust based on the clinician’s critical thinking skills leveraged to discern value of machine outputs within clinical context. Utilization of collaborative intelligence should be staged with the level of understanding and evidence. It is particularly well suited to low-complexity non-urgent care and to identifying individuals at rising risk within a population. Clinician involvement in algorithm development and the amassing of evidence to support safety and efficacy will propel adoption. Utilization of collaborative intelligence represents the natural progression of health care innovation, and if thoughtfully constructed and equitably deployed, holds the promise to decrease clinician burden and improve access to care.

## Vision of collaborative intelligence

Collaborative intelligence is the use of advanced analytics and computing power with an understanding that humans bear responsibility for the accuracy, completeness and any inherent bias found in the training data^1^. Artificial intelligence refers to when computer systems perform tasks that normally require human intelligence^2^. In collaborative intelligence, human users work in concert with algorithms, interpreting artificial intelligence outputs, with the intention of together, becoming more intelligent. In the next 12 to 18 months, collaborative intelligence will transform clinical data management via the analysis of millions of clinical data points. Radiology has already been transformed by this technology and the advent of collaborative intelligence will allow the ingestion of information beyond imaging to be integrated into clinical care across multiple specialties^3^. Importantly, collaborative intelligence is different than human-in-the-loop machine learning, which refers to leveraging the rapid detection and computing power of AI while having humans involved in the algorithm development process to fill in gaps where machine learning may fall short. Therefore, human in the loop utilizes humans in the service of improving the algorithm. Conversely, collaborative intelligence is the use of algorithms in the service of improving, but not replacing, human acumen.

We see collaborative intelligence as part of a compelling vision of the near future of healthcare where clinical acumen interacts with technology to finally optimize the care of patients. Here we share the definition of collaborative AI, why it has emerged, the challenges it poses, and how we harness its power. We present a theory for how physician responsibility and interactivity with technology can yield trust and create a roadmap of the future for how these technologies present in practice. We offer a taxonomy of applications/technology, which might serve as shorthand for how/when/why clinicians should trust them. Collaborative intelligence offers a novel philosophy of how medicine can embrace data technologies and has the potential to be developed into a true method for approaching the digital transformation of healthcare while keeping the clinician-patient relationship at the center of the novel pyramid of care delivery.

## Why Collaborative Intelligence has emerged

Why adopt collaborative intelligence? The increasing burden of disease^4^ cannot be managed in a one-to-one relationship by the decreasing clinical workforce^5,6^. The current healthcare challenge has three components: accelerating complexity and exponential information overload^7^, a transition to continuous care models and optimized resource allocation^8^, and a mandate to eradicate disparities in access and quality^9^. Implementing data analytics and artificial intelligence addresses these challenges and optimizes patient care.

## Addressing accelerating complexity and exponential information overload

Patient data has historically been disparate and disconnected in a clinical setting. Patient-generated data from multiple sources compounds this problem. We have a greater opportunity than ever before to create a comprehensive picture of a patient, but that can only be realized with the application of AI to assemble relevant patient and population data to achieve high-quality care^10^. To create the infrastructure necessary to utilize collaborative intelligence effectively and ethically, it is imperative to systematically create standardized and transparent data. Collaborative intelligence increases the value of electronic health records via summarization, risk prediction and pattern recognition, and data abstraction for quality metrics and registries^11^. Clinical decision support harnesses the power of patient level data and guidelines to optimize care. With the sheer volume of data—patient-reported outcomes, wearables, medical-grade remote monitoring, soon perhaps ambient continuous data, combined with the rapid pace of scientific advances, guidelines, and trials—delivering the highest quality care remains in reach only with support from advanced computing^1^. Many health organizations have moved beyond harvesting data solely from health records and toward the creation of institutional data lakes that link a variety of clinical and operational datasets, all in pursuit of better care and operational performance^12^. Such data aggregation provides more opportunity to apply AI-enabled solutions. Incorporation of social determinants of health data further supports clinicians and leading health systems in human-centered design of solutions to increase access to and continuity of care. To best manage these multiple streams of patient information, data analytics are essential to the future practice of healthcare.

Once the above-mentioned data are assembled, the scientific advances of the last several decades can now be incorporated into treating patients with the full rigor available in the literature. Data analytics and collaborative intelligence offer the unique opportunity to elevate the foundation of medical care and apply scientifically rigorous clinical knowledge during the clinician patient encounter. Guideline directed medical therapy (GDMT), although available, has yet to reach its full potential as well^13^. With the increasing burden and complexity of disease^14^ and a declining clinical workforce^6,15^, operational sustainability demands engagement with AI to routinely achieve appropriate standards of care and offer novel innovative treatments for patients under our care.

To address the rising administrative burden in healthcare, generative AI deserves special comment. Generative AI refers to the use of large language models that read and search text to produce textual results to focus questions. The uses of generative AI in medicine abound, but will start with administrative tasks like documentation, letters, forms, and reminders whether via electronic health records, independent applications or chatbots, but may eventually be useful as an improved search tool for clinical information at the point of care to enhance clinical care. While safety and responsibility are essential to building this framework for generative AI use in clinical medicine, there is a role for ongoing pilot studies to better assess the capabilities and risks of generative AI.

This is unique for a technology, because unlike prior technology advances, generative AI needs to be used for the technology to evolve. Until now, we have been cautious to not let consumers or lay people see “unfinished” tech work. However, the field of technology is changing. What we want from ChatGPT and other generative AI models is the truth, however, if we do not allow technology use in the real world, we cannot iterate on it to make it “safe”. Therefore, an understanding that AI can serve as a collaborator in helping us investigate new ideas, compose thoughts on a subject, plan a dinner party, or create new hypotheses to test is what creates the power of the technology. Its power lies not in the massive amounts of computing and assessment it can do, rather in its ability to offer that up for human intelligence to assess and then … the real test is whether it can successfully integrate the new information and iterate its own processes. The only way to gauge the power of these technologies is to use them while recognizing our responsibility to design and test its use for exponential information overload with the lens of collaborative intelligence.

## Continuous care and optimized resource allocation

Episodic clinical care does not align with patients’ continuous experience of disease. Asynchronous communication with patient reported outcome measures and patient portals allow for free-flowing communication between clinical teams and patients. Blended care, the combination of in person and virtual synchronous visits, whether by telephone or video, increases the convenience of care, decreases the cost of obtaining medical care, and allows individuals to consume care in non-threatening environments^16^. The wide adoption of digital tracking and wearables and increased availability of medical grade devices provides essential support for screening, monitoring, and treatment for effective remote cardiovascular care. Lastly, patients engage with digital devices and present data to clinicians for interpretation and follow up.

Technology enabled care, including digital health technology with “AI inside,” expands the reach of a limited number of clinicians to the communities where people live and, if thoughtfully deployed, improves health equity through greater access to standardized high-quality care^17^. The management of chronic disease using AI-enabled digital screening mechanisms supports earlier identification of disease progression, optimizing clinical workflows to serve patients in a timely manner with the appropriate resources^18^. Those individuals who need chronic management can be managed at home using many of the systems just discussed. This allows patients to partner in their care while remaining local. Additionally, when these systems recognize that individuals have rising risk, and analytic infrastructure can identify potential progression of illness, patients can be brought to attention more promptly^19^. In those situations where patients cannot be managed with remote or virtual care, or require intervention at tertiary centers, the mechanism of utilizing AI allows clinicians to have the knowledge to orient patients to the right next test, correct clinical team, and most appropriate location of care^17^. In this manner, continuous analysis of data offers population health feedback, earlier screening and diagnosis and appropriate resource utilization for the most urgent or complex patients.

## Mandate to eradicate disparities in access and quality

Population health analytics enable clinicians to understand the burden of disparities within a community^20^. Linked datasets, as described above, for individual patients can also be linked across populations of patients. AI then becomes a powerful tool for identifying patterns within those datasets, including assessment of outcomes within the group for the purpose of identifying outliers; adherence to GDMT; and, where appropriate, adherence to guidelines specific to certain communities with unique risk profiles^20^. Understanding outcomes supports the replication of best practices and allows interrogation of the underlying causes of poorer outcomes. Detecting patients who fall out of alignment with GDMT becomes the first step in closing gaps. Finally, this suite of tools allows for a closer look at subpopulations at elevated risk for disease based on demographic, ethnic or lifestyle factors, to bring them into better clinical management^21^.

For these systems to work optimally, we must assure equitable access to technologies at the individual level. Digitally enabled care can include direct access to technology and access to the benefits of technology-enhanced data analytics and population health efforts. Access at the individual level in underserved communities is complex and multifaceted. It will require interventions including improved connectivity, devices, and access to low-tech solutions that allow the delivery of virtual care without local infrastructure. In designing for digitally enhanced care, caregivers must not assume that certain communities cannot utilize devices or technology^21^. While system level access is often necessary for digital adoption at the community level, broadband access is not the only impediment to engaging with technology^22,23^. Lower tech solutions have a role to play in strengthening access and should not preclude the ability to benefit from insights derived from data^22^.

Finally, for health systems to use the power of AI to scale care delivery without amplifying inequities, we must have a framework to perform active surveillance for bias^24^. The development of software for AI demands fairness, operational tolerances, and surveying for reinforcement of systemic bias^24^. When these mechanisms involve an assessment of influences on vulnerable populations, we can limit bias upstream^24^. Everyone involved in the process has a responsibility to promote ethical models, including clinicians, health systems, vendors in the technology sector and regulatory authorities^24^. Importantly, recognizing that social determinants of health are not static and may change over time will require iterative assessment^24^. Infrastructure will then be designed to be agile and contextually relevant.

## Physician responsibility and interactivity with technology

For the promise of collaborative intelligence to be in reach, clinicians must become active in understanding the role of algorithms and artificial intelligence in healthcare. A myriad of solutions already exist in market, used either directly by the consumer, large companies in healthcare, or early adopters^22^. There has been, and will continue to be, a rapid rise in novel technologies which utilize collaborative intelligence to increase the accuracy and power of disease detection and risk stratification. The digital transformation of healthcare using AI demands that clinicians offer rapid feedback to ensure safety. The systematic interaction between clinical practitioners and AI in the construct of collaborative intelligence provides critical inputs to maintain safe, high quality, optimal patient care as the pace of healthcare innovation accelerates.

Successful implementation of these technologies will fail without trust. A paradox exists in clinical care today: technology has played an assistant role in health care for decades, but patients and end users experience reluctance in using AI in care^23^. To mitigate the risk of blindly following computing outputs, AI-enabled clinicians must understand their responsibility in ethical use of AI. AI should not be evaluated in a vacuum, but instead within the context of the use of the technology by the clinician, system, or business. To gain the trust required for broader adoption, collaborative intelligence depends on responsible use of data and algorithms, an AI-enabled workforce, and transparency around technology’s limitations.

Critical thinking skills must be deployed to evaluate the AI-sourced and created information and make informed decisions. Clinicians and hospitals require tools and resources to verify information, such as links to credible sources, to continuously monitor and train AI models and correct inaccuracies or biases as needed^22^. Vast real-world datasets play an essential role to train accurate AI models and retrain as needed. AI-generated responses can be labeled such that users understand the need to validate AI’s findings^22^.

As AI in clinical decision making evolves, explainability is touted as one mechanism to increase transparency and trust in AI. Explainable machine learning offers insights into the behavior of AI models. Although machine learning engineers use a variety of techniques to understand how a model behaves and improve upon it, in its current form, explainable AI is not designed to be used by clinician end users at the point of care. Until there are organizational frameworks for deciding when we need an explanation, and why, explainable machine learning remains most useful to the engineers creating the AI models.

## Clinical applications of collaborative intelligence

The implementation of collaborative intelligence will evolve as clinicians gain trust and systems create infrastructure. Clinicians need to understand what part of the process or narrow outcome the AI aims to address. Interpretability and familiarity with collaborative intelligence systems helps users learn the system, but also helps the system align better with clinical intuition. Clinicians require both experiential and didactic training to appropriately engage with AI in clinical care. The wisdom to “start small” applies well in this context. Users will develop appropriate trust in these systems once they utilize collaborative intelligence for low complexity, non-urgent care and understand how the systems make their decisions. With success in low complexity and nonurgent settings, attention can be turned to higher risk and more urgent matters.

AI-driven health solutions in practice have proven more efficient and effective, though the challenge remains in scaling up these technologies. For example, a study of safety and efficacy of AI for left ventricular ejection fraction assessed AI decision support vs sonographer reading on timing and accuracy of echo assessment. Designed as a randomized single group assignment of 3,495 patients, the researchers found that cardiologists needed to revise interpretations more often with sonographer assessed scans (27.2% of the time) compared to the need to change the report with AI assessed initial scans (16.8% of the time)^25^.

To create collaborative intelligence that provides clinical support at the point of care, there must be clinicians involved in algorithm development and peer-reviewed clinical guidance must be incorporated. The clinician’s acumen may be challenged at times as the AI notes new trends, data, or research, but at other times, their nuanced knowledge will supersede the AI’s ability. If the AI-enabled clinician understands that both scenarios are within the realm of appropriate responses, they can engage in process improvement to iterate and improve upon the technology. Iterative interaction enables further training of algorithms, supports unlearning parts of AI that do not fit a given situation, identification of bias or incorrect assumptions, and learning about novel groupings which may lead to clinical insights^1^. Engaging with collaborative intelligence requires AI awareness, not expertise. Importantly, if employing collaborative intelligence in the clinical arena, human design centered processes are essential to create seamless experiences for already overwhelmed clinicians.

At the population level, the application of collaborative intelligence in practice aids in identifying rising risk individuals to direct them to timely care in the appropriate location. In both the outpatient and inpatient setting, data-driven collaborative intelligence can help meet quality and safety measures such as response times to changing patterns^26^. Finally, AI has the power and speed that surpasses the human brain. The clinician guides care processes at the individual and population level by reviewing the continuously improving analytics offered by collaborative intelligence and guiding its direction as a conductor would a symphony. Final implementation of care always lies with the clinical team with shared decision making with the patient.

The study of the impact and limitations of AI in care is an essential ultimate step in the cycle of application of collaborative intelligence. Safe and high-quality outputs from AI require the developer and end user to agree upon and define acceptable margins of error for collaborative intelligence models since the clinician enabled by AI computing power can amplify success as well as errors. Observed efficiencies and benefits to one group of patients may adversely affect other segments of the population or system, therefore measuring outputs requires a complex systematic review of downstream effects and an infrastructure for iterative assessment and correction. Openly stating the limitations of digital data and AI in healthcare can help breed trust in new systems just beginning to prove their ability to meet basic healthcare competency.

## Conclusion

Thoughtfully created and equitably deployed collaborative intelligence is a natural progression of innovation in healthcare. The dichotomous concept of technology vs technology-free care has not existed in practice. Clinicians regularly engage with technology while providing patient care. Therefore, technology should not be evaluated on its own, but instead within the context of the clinical environment, system, or business in which it operates. AI holds the promise to transform the ability of humans to interpret substantial amounts of data which the brain has not previously been able to process. AI enabled improvements to accessing information create efficiencies in clinical practice and empower patients with more curated information.

Neither a human brain nor an AI algorithm can achieve perfect accuracy and precision. Human operators can program systems with unbiased, fair data to generate insights and then use individual judgement to decide what comes next. End users must acknowledge the promise and limits of AI, recognize it will never have a moral compass, and take responsibility for the data used to train algorithms while interpreting the outputs fairly. Standards governing the use and protecting from misuse and harm resulting from this technology must be developed and used in clinical care. By recognizing the limitations of AI-enabled technologies, clinicians and patients can unlock the helpful use cases for these technologies to support better quality, more equitable care.

## Data Availability

Not applicable.
